# No skin off your back: the sampling and extraction of sebum for metabolomics

**DOI:** 10.1007/s11306-023-01982-3

**Published:** 2023-03-24

**Authors:** C. Géhin, J. Tokarska, S. J. Fowler, P. E. Barran, D. K. Trivedi

**Affiliations:** 1 School of Chemistry, Manchester Institute of Biotechnology, University of Manchester, Princess Street, Manchester, M1 7DN UK; 2 Department of Respiratory Medicine, Manchester University Hospitals NHS Foundation Trust, Manchester, UK; 3 Faculty of Biology, Medicine and Health, School of Biological Sciences, University of Manchester, Manchester, UK; 4 NIHR Manchester Biomedical Research Centre, Manchester University Hospitals NHS Foundation Trust, Manchester, UK

**Keywords:** Sebum, Biomarkers, Sebum extraction, Sebum enrichment, Metabolomics, Lipidomics

## Abstract

**Introduction:**

Sebum-based metabolomics (a subset of “sebomics”) is a developing field that involves the sampling, identification, and quantification of metabolites found in human sebum. Sebum is a lipid-rich oily substance secreted by the sebaceous glands onto the skin surface for skin homeostasis, lubrication, thermoregulation, and environmental protection. Interest in sebomics has grown over the last decade due to its potential for rapid analysis following non-invasive sampling for a range of clinical and environmental applications.

**Objectives:**

To provide an overview of various sebum sampling techniques with their associated challenges.

To evaluate applications of sebum for clinical research, drug monitoring, and human biomonitoring.

To provide a commentary of the opportunities of using sebum as a diagnostic biofluid in the future.

**Methods:**

Bibliometric analyses of selected keywords regarding skin surface analysis using the Scopus search engine from 1960 to 2022 was performed on 12th January 2023. The published literature was compartmentalised based on what the work contributed to in the following areas: the understanding about sebum, its composition, the analytical technologies used, or the purpose of use of sebum. The findings were summarised in this review.

**Results:**

Historically, about 15 methods of sampling have been used for sebum collection. The sample preparation approaches vary depending on the analytes of interest and are summarised. The use of sebum is not limited to just skin diseases or drug monitoring but also demonstrated for other systemic disease. Most of the work carried out for untargeted analysis of metabolites associated with sebum has been in the recent two decades.

**Conclusion:**

Sebum has a huge potential beyond skin research and understanding how one’s physiological state affects or reflects on the skin metabolome via the sebaceous glands itself or by interactions with sebaceous secretion, will open doors for simpler biomonitoring. Sebum acts as a sink to environmental metabolites and has applications awaiting to be explored, such as biosecurity, cross-border migration, localised exposure to harmful substances, and high-throughput population screening. These applications will be possible with rapid advances in volatile headspace and lipidomics method development as well as the ability of the metabolomics community to annotate unknown species better. A key issue with skin surface analysis that remains unsolved is attributing the source of the metabolites found on the skin surface before meaningful biological interpretation.

## Introduction

The examination of bodily excretions has been a part of medical investigation long before the term “metabolomics” was coined. For example, urinalysis was used as a diagnostic tool 6000 years ago (Armstrong, 2007), where Sumerian and Babylonian physicians recorded their pathological assessments of colour and consistency on clay tablets (Wellcome, 1911). Nowadays, due to advances in technology, modern diagnostics typically employ metabolomics to probe into a biological sample’s chemical composition. Metabolomics is an analytical profiling technique that generally uses hyphenated mass spectrometry and bioinformatics to determine the number and concentration of metabolites in biological samples. It has risen in prominence as a field that comprises both biomarker discovery and molecular diagnostics.

Metabolomics assays can be applied to a wide range of biological samples, where the choice of the sample should be driven by the clinical question being investigated. It is common to study biofluids to discover potential diagnostic biomarkers; whereas, the use of tissues or cells has more application in investigating physiological mechanisms (Chetwynd et al., 2017). A bibliometric analysis of biofluids used for metabolomics research in 2021 revealed blood, urine, and faeces to be the most popular with 55.1%, 12.2%, and 10.8% of total published papers using them, respectively.^1^ Despite this, there is a growing interest in the metabolomics of less conventional biological matrices, one of them being skin secretions. Skin secretions are a mixture of sebum, sweat, corneocyte debris, and proteolytic products of filaggrin, collectively known as “residual skin surface components” (RSSC) (Dumas & Ntambi, 2018; Ludovici et al., 2018; Shetage et al., 2018). Their composition is predominantly governed by complex hormonal and metabolic mechanisms and may be confounded by interactions with the external environment and bacterial colonies on the skin surface (Bolognia et al., 2018; Bouslimani et al., 2015; Dumas & Ntambi 2018; Lovászi et al., 2017; Luca & Valacchi, 2010; Zouboulis et al., 2016). Therefore, they can provide a wealth of information regarding the body’s physiological state. Skin secretions are readily available as superficial fluids and thus offer an attractive opportunity for developing non-invasive diagnostic tests with point-of-care potential.

Initially, the interest in RSSC as a medium for disease diagnostics started due to body odour (Kippenberger et al., 2012). Olfactory diagnosis is not novel and has been used in the past for diseases, such as scurvy, schizophrenia, smallpox, and typhoid (Penn & Potts, 1998). The chemicals typically responsible for these characteristic body odours are volatile organic compounds (VOCs). Skin VOCs are predominantly synthesised by either one’s internal metabolism, bacterial activity on the skin surface, exogenous deposition, or skin reactions with environmental factors, such as ozone and UV (Akitomo et al., 2003; D’Orazio et al., 2013; Drakaki et al., 2014; Lacy et al., 2014; Li et al., 2021; Luca & Valacchi, 2010; Natsch & Emter, 1800; Wisthaler & Weschler, 2010). Although VOCs can originate from both sebaceous and sweat glands, the excretion from the latter has been studied for odour-based metabolites more often (Brasier & Eckstein, 2019; Gallagher et al., 2008). Biomarkers for several diseases, such as diabetes (Provitera et al., 2010), lung cancer (Calderón-Santiago et al., 2015), schizophrenia (Raiszadeh et al., 2012), and cystic fibrosis (Carter et al. 1984; Vinayavekhin et al., 2010), have already been identified in sweat. As VOCs span a wide range of polarities, the hydrophobic VOCs would be more likely to partition into sebum than sweat and vice versa; therefore, sebum should equally be seen as a potential reservoir of odorous compounds. This is supported by recent studies involving Parkinson’s disease that have discovered odorous hydrophobic volatile biomarkers in sebum (Fu et al., 2022; Trivedi et al., 2019).

**Fig. 1:**
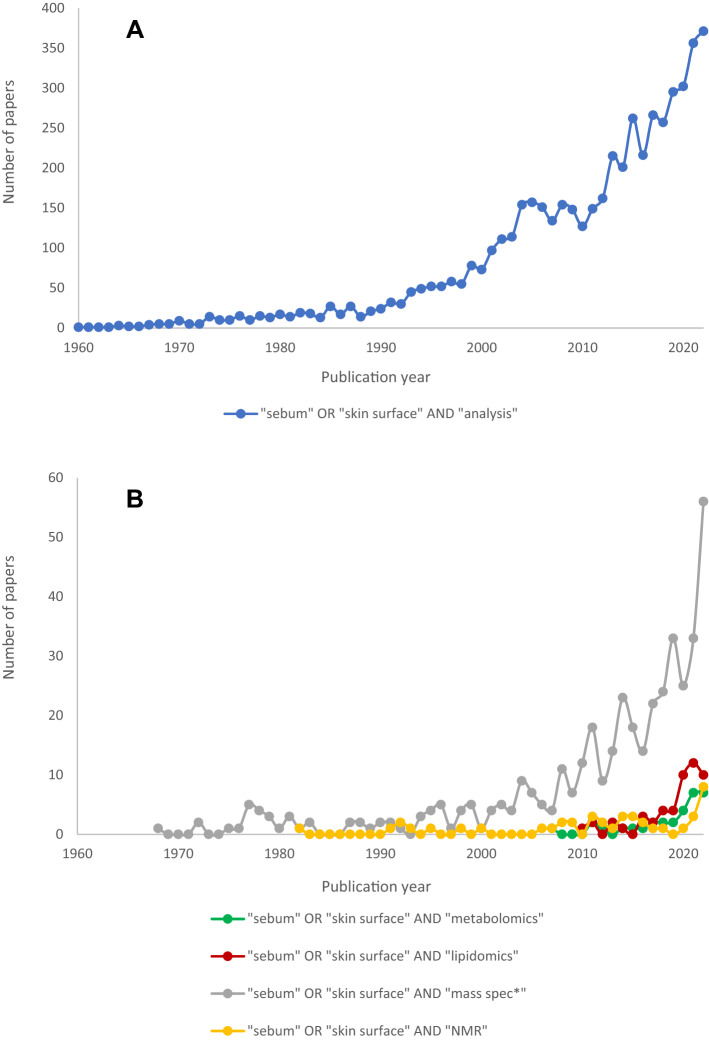
Bibliometric analyses of selected keywords regarding skin surface analysis using the Scopus search engine from 1960 to 2022 (performed on 12th January 2023). The exact search phrases are presented in the plot legends

Sebum as a biofluid has been investigated for many decades with a focus on understanding normal and pathological skin biology. A multitude of papers originating in the 1950–1980s form the basis of sebum knowledge concerning composition and production. This predates metabolomics and the associated advanced mass spectrometric technology that has enabled more robust analyte characterisation and metabolome screening capabilities. Bibliometric analyses of literature ranging from 1960 to 2022 demonstrate the increasing popularity of skin surface analysis over time (Fig. 1), while Fig. 1B explicitly demonstrates the novelty of this biofluid for metabolomics and lipidomics as less than five papers were published in either discipline until 2020. The slow adoption of sebum is likely due to the technological limitations associated with the difficulties of reliable, consistent sebum sampling, the challenges of analysing complex biological matrices for biomarkers, and the inability of chemical detectors to accurately identify and quantify low concentration compounds.

The novelty and ease of sebum analysis and its potential to direct clinical testing have recently attracted worldwide interest, particularly for Parkinson’s disease (Quiqley, 2019). With sebum research in its infancy, there is a lack of standardised protocols for sebum sampling and extraction, i.e., the front end of an analytical procedure. The subsequent back end of the procedure involving data acquisition and interpretation can be applied as usual using well-established routine metabolomics workflows (Alonso et al., 2015; Alseekh et al., 2021; Ashrafian et al., 2021; Cui et al., 2018; Dudzik et al., 2018; Nalbantoglu et al., 2019; Rakusanova et al., 2023; Sarandi et al., 2021; Schrimpe-Rutledge et al., 2016; Segers et al., 2019; Wishart, 2019). Metabolomics studies rely on the quality of sample heavily; as the saying goes: “rubbish in, rubbish out”. This is truly applicable for sebum analysis because, unlike biofluids such as urine and blood that have more straightforward sample collections, there are many diverse sebum sampling techniques currently available that all collect metabolites differently. This selectivity means that without prior knowledge, a researcher would be subject to a systemic bias from their sampling medium that will affect their research outcomes. Therefore, this review summarises our current understanding of sebum composition and the state-of-the-art technologies globally used for sebum sampling. Finally, an evaluation of the applications of sebum is performed regarding clinical research, drug monitoring, and human biomonitoring, to provide a commentary of the opportunities of using sebum as a diagnostic biofluid in the future.

## What is sebum?

**Fig. 2:**
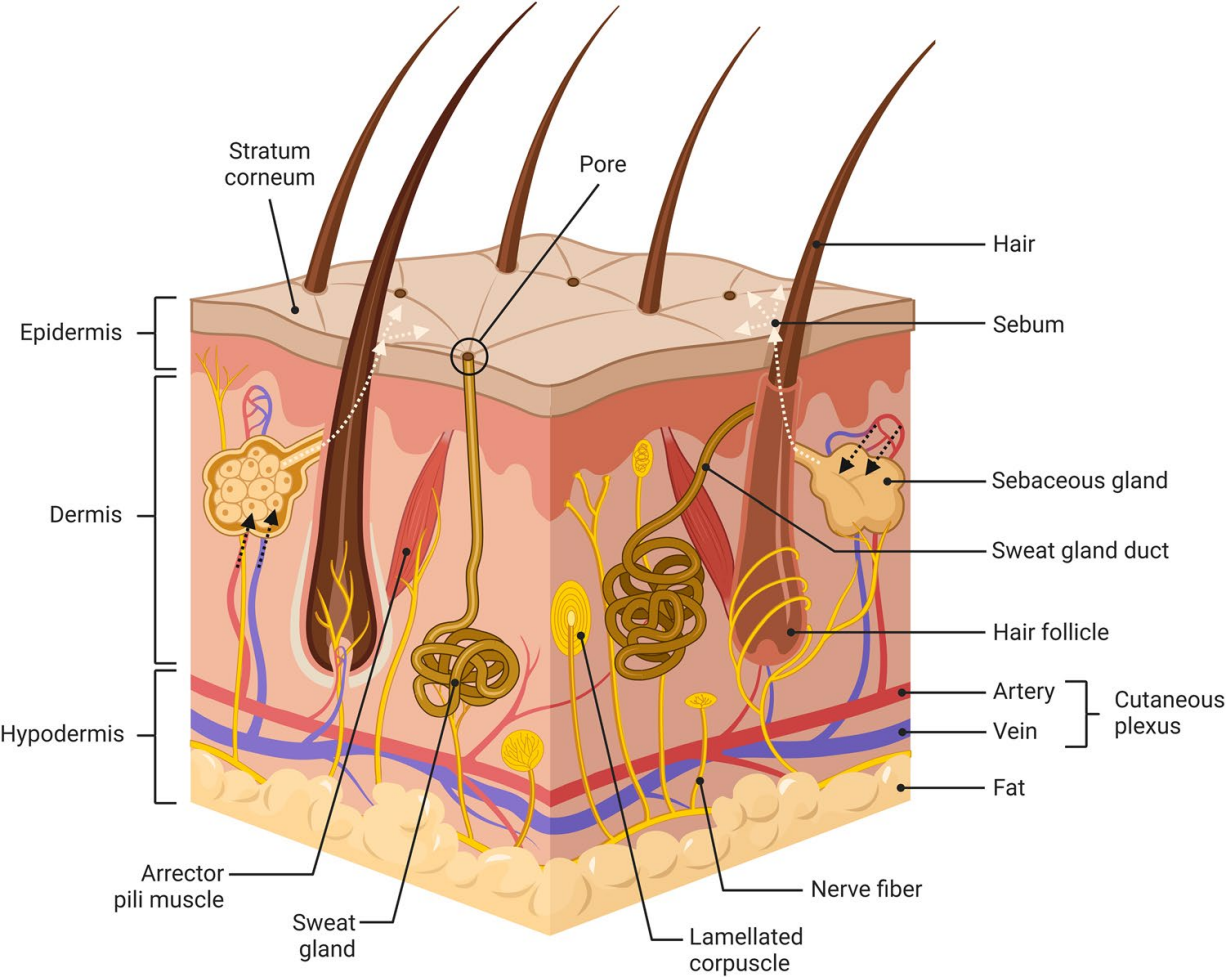
Skin structure adapted from “Anatomy of the Skin” by BioRender.com (2022). Retrieved from https://app.biorender.com/biorender-templates. The dashed light-yellow lines show how sebum travels from the sebaceous gland to the skin surface. The dashed black lines represent the uptake of preformed lipids by the sebaceous glands from blood circulation

Sebum is a light yellow, lipid-rich fluid produced by the sebaceous glands through the holocrine secretion of sebocytes (Firooz et al., 2015; Honari et al., 2014; Zouboulis et al., 2016), the major cells within the sebaceous glands (Niemann & Horsley, 2012; Zouboulis et al., 2016). After production, sebum is discharged into the sebaceous duct and then travels along the hair follicle onto the skin surface (Fig. 2) (Nicolaou & Harwood, 2016). This process takes approximately 2–3 weeks (Nicolaou & Harwood, 2016). The role of sebum in skin barrier function is not completely understood; however, it is generally accepted as key in maintaining skin homeostasis, lubrication, thermoregulation, and protection from environmental stressors, pathogens, and contaminants by producing either proinflammatory or anti-inflammatory cytokines, chemokines, interleukins, pheromones, free fatty acids, and hormones (Dumas & Ntambi, 2018; Honari et al., 2014; Luca & Valacchi, 2010; Nicolaou & Harwood, 2016; Niemann & Horsley, 2012; Picardo et al., 2015; Shamloul & Khachemoune, 2021; Smith & Thiboutot, 2008).

Sebum can be found all over the body except for the palms of the hands and soles of the feet due to the lack of sebaceous glands there (Borda & Wikramanayake, 2015; Smith & Thiboutot, 2008). The largest number of glands and the most sebum-rich parts of the body are the face (T-zone), back, and upper chest, where the number of glands ranges from 400 to 900 glands/cm^2^ (Borda & Wikramanayake, 2015; Smith & Thiboutot, 2008; Thody & Shuster, 1989). The rate of sebum production varies between individuals due to many factors, such as sex, age, ethnicity, diet, temperature, and circadian rhythm (Table 1). These are all important aspects to consider for sampling purposes and in the experimental design. Generally, over three hours, a rate of less than 0.5 mg/10 cm^2^ is associated with dry skin (Bolognia et al., 2018), approximately 1 mg/10 cm^2^ is the adult average (Plewig & Kligman, 2000), and 1.5–4.0 mg/10 cm^2^ is associated with seborrhoea (Bolognia et al., 2018).

**Table 1:** Demonstrated effects of variables on sebum production and sebum composition

Variable	Effect on sebum production	Effect on sebum composition	Molecules impacted	References
Sex	↕	↕	Di/triacylglycerols, fatty acids, squalene, wax esters, cholesterol	Shetage et al. (2018), Cotterill et al. (1972a), Shetage et al. (2014), Man et al. (2009), Roh et al. (2006) and Jacobi et al. (2005)
Hormone—testosterone ↑	↑	↕	Squalene, wax ester, cholesterol	Bolognia et al. (2018), Cotterill et al. (1972a), Pochi and Strauss (1969) and Giltay and Gooren (2000)
Hormone—oestrogen ↑	↕	↕	Triacylglycerols, cholesterol	Bolognia et al. (2018), Cotterill et al. (1972a), Roh et al. (2006) and Giltay and Gooren (2000)
Menstrual cycle	↕	X	N/A	Roh et al. (2006) and Piérard-Franchimont et al. (1991)
Age	↕	↕	Triacylglycerols, fatty acids, squalene, wax esters, cholesterol esters, cholesterol	Shetage et al. (2018), Cotterill et al. (1972a), Shetage et al. (2014), Man et al. (2009), Marrakchi and Maibach (2007), Wilhelm (1991), Pappas et al. (2013), Sansone-Bazzano et al. (1980), Ramasastry et al. (1970) and Qiu et al. (2011)
Ethnicity	↕	↕	Diacylglycerols, fatty acids, squalene, wax esters	Shetage et al. (2018), Shetage et al. (2014), Pappas et al. (2013), Rawlings (2006), Rebora and Guarrera (1988) and Warrier et al. (1996)
Diet—excess fats/carbs	↑	↕	Triacylglycerols, fatty acids, wax esters	Agache et al. (1980), Kim et al. (2010), Wilkinson (1966) and Macdonald (1964)
Diet—calorie deprivation	↓	↕	Di/triacylglycerols, fatty acids, wax esters, cholesterol esters, cholesterol	Downing et al. (1972) and Pochi et al. (1970)
Temperature ↑	↑	↕	Squalene	Qiu et al. (2011), Piérard-Franchimont et al. (1990), Youn et al. (2005) and Williams et al. (1973)
Circadian rhythm—day to night	↓	X	N/A	Verschoore et al. (1993), Burton et al. (1970) and Fur et al. (2001)
Seasonal variations—winter to summer	↑	X	N/A	Qiu et al. (2011), Piérard-Franchimont et al. (1990) and Youn et al. (2005)
Anatomical site	↕	↕	Triacylglycerols, fatty acids, squalene, cholesterol	Ludovici et al. (2018), Marrakchi and Maibach (2007), Wilhelm (1991), Youn et al. (2005), Oh et al. (2014), Sheu et al. (1999), Stevens et al. (2015), Greene et al. (1970) and Cho et al. (2021)

X = no significant changes observed/lack of studies; ↑ = increase; ↓ = decrease; ↕ = significant changes observed/conflicting observations between research groups

**Fig. 3:**
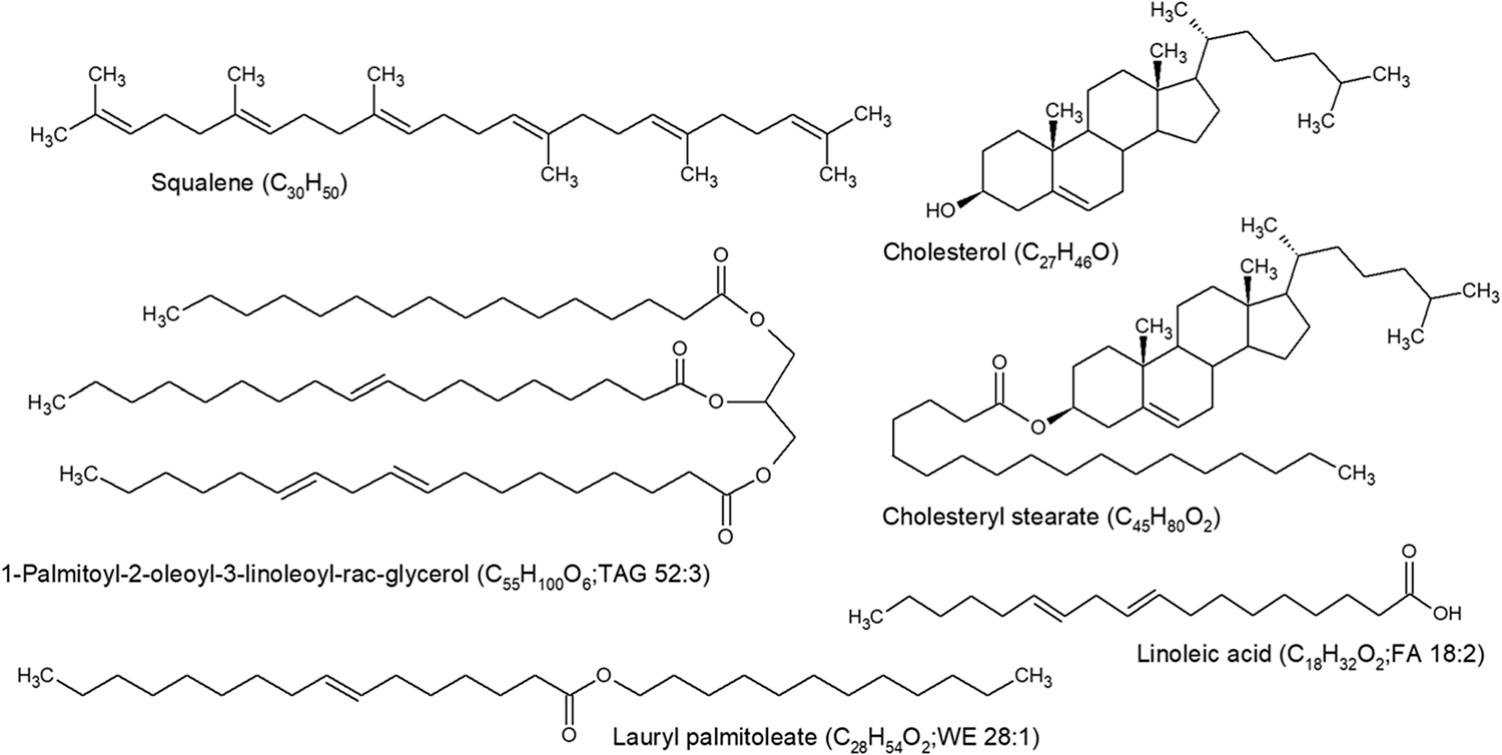
Example compounds present in human sebum of the different classes: triacylglycerol (TAG), fatty acid (FA), squalene, wax ester (WE), cholesterol, and cholesterol ester

The relative composition of sebum is 30–50% triacylglycerols/diacylglycerols, 15–30% free fatty acids, 12–20% squalene, 26–30% wax esters, 3–6% cholesterol esters, and 1.5–2.5% cholesterol (Fig. 3) (Picardo et al., 2009; Smith & Thiboutot, 2008). With most logPs > 5 (Aldana et al., 2020), these compounds are predominantly hydrophobic with little/no evidence in literature, to the best of our knowledge, of hydrophilic metabolites in sebum. It should be noted that the sebum composition observed is dependent on the sampling method used. For instance, it is known that sebaceous triacylglycerols undergo partial hydrolysis into free fatty acids and diacylglycerols by bacterial lipases; therefore, sampling before or after this hydrolysis would impact the overall composition (Downing et al., 1969; Wertz, 2018). These sebaceous lipids are produced through dermal substrate-enzyme reactions and uptake from blood circulation. The composition of human sebum differs markedly compared to other mammalian species (Nikkari, 1974; Picardo et al., 2009; Smith & Thiboutot, 2008; Stewart et al., 1991).

The most characteristic products of sebum are squalene and wax esters. They are almost exclusively found in sebum, although small amounts of them have been detected in saliva (Brasser et al., 2011; Picardo et al., 2009). Squalene is an intermediate in the biosynthetic pathway producing cholesterol. In the sebaceous gland, the completion of this process is halted as squalene does not undergo further transformation into lanosterol. Due to the uniqueness of its accumulation in sebum, it may be considered a marker for sebocyte differentiation and thus for sebum production (Picardo et al., 2009). It is worth noting that squalene has been shown to rapidly oxidise due to a lack of dietary vitamin E or through exposure to sunlight, cigarette smoke, dust, ozone, and other air pollutants (Curpen et al., 2020; Pham et al., 2015; Stefaniak et al., 2010).

Many studies have shown that sebum composition and production vary between individuals (“inter-variability”) but are relatively constant within individuals (“intra-variability”) (Downing et al., 1969; Green et al., 1984). Differences in sebum composition have been attributed to (but not limited to) sex, age, ethnicity, diet, and hormones (Table 1). Sebum profiles also change depending on what anatomical site it is sampled from due to the different volatile compounds, bacterial, fungal, and viral colonies present (Table 1). This literature review showed a lack of studies of the effects of circadian rhythm, menstrual cycle, seasonal variation, and fasting on sebum composition (Table 1). The degree to which all of these factors influence sebum is poorly understood and requires additional work to understand the baseline sebum to allow any meaningful clinical interpretation of results.

Studying the correlation between sebum and blood could prove invaluable in physiological understanding and biomarker discovery. The sebaceous glands express the fatty acid transport protein (FATP) and low-density lipoprotein (LDL) receptors that are responsible for the uptake of lipids from blood circulation for the hypothesised elimination via sebum secretion (Fig. 2) (Dumas & Ntambi, 2018; Shetage et al., 2018; Villas-Bôas et al., 2011; Zhou et al., 2012). This lipid elimination is supported by several experimental observations; for instance, a diet high in fats and carbohydrates causes an increase in sebum production (Kim et al., 2010; Macdonald, 1964; Pochi et al., 1970; Wilkinson, 1966), the incorporation of free fatty acids into sebum is reduced by 20% at the beginning of fasting (Downing et al., 1972; Pochi et al., 1970), and that inhibition of sebum production using isotretinoin resulted in significantly increased plasma triacylglycerol and cholesterol levels (Bershad et al., 1985; Zech et al., 1983). This suggests that there is the potential that biomarker trends previously identified in blood could be similarly observed in sebum.

## Current sampling and extraction procedures

Sebum sampling has a long history with early scientific studies in circa 1910 extracting skin lipids from long johns worn for extended periods (Strauss & Pochi, 1961). Since then, a multitude of non-invasive sebum sampling techniques have been developed with their relative advantages and disadvantages (Table 2). Sebum is sampled either directly from the skin or from the headspace over the skin. For untargeted profiling, it is ideal for the sampling process to be chemically unselective to collect the largest number of analytes and generate the most comprehensive sample signature. Unfortunately, due to the diversity in structure, polarity, and volatility of the compounds present, this is not possible using a single sampling method and these sampling biases should be considered in the study design and the interpretation of results.

**Table 2:** Summary of non-invasive sampling techniques used for sebum analysis

	Sampling technique	Target analytes*	Home sampling potential	Speed (min)	Cost (per sample)	Ease of use	References
Direct contact	Solvent extraction	ALL**		< 5	$		Marrakchi and Maibach (2007), Wilkinson (1966), Greene et al. (1970), Downing et al. (1969), Cunliffe et al. (1971), Cotterill et al. (1972b), Clarys and Barel (1995), Piérard et al. (2000), Millns and Maibach (1982), Emanuel (1936), Jones et al. (1951) and Afghani et al. (2021)
	Glass plates/beads	ALL		< 5	$$		Agache et al. (1980) and Eberhardt and Trieb (1979)
	Bentonite clay	ALL		180	$		Clarys and Barel (1995), Piérard et al. (2000), Downing et al. (1982) and Stewart and Downing (1985)
	Absorbent papers (e.g., cigarette papers)	ALL		20–180	$		Shetage et al. (2018), Shetage et al. (2014), Ramasastry et al. (1970), Williams et al. (1973), Burton et al. (1970), Strauss and Pochi (1961), Cunliffe et al. (1971), Cotterill et al. (1972b), Clarys and Barel (1995), Piérard et al. (2000), Cotterill et al. (1971), Michael-Jubeli et al. (2011), Michael-Jubeli et al. (2018), Cunliffe and Shuster (1969), Pochi et al. (1970) and Motoyama and Kihara (2017)
	Sponge	ALL	✓	5–180	$		Shetage et al. (2014), Ramasastry et al. (1970) and Cunliffe et al. (1971)
	Silica plates	ALL	✓	< 5	$		Esteves et al. (2018), Lima et al. (2015) and Delafiori et al. (2021)
	Cotton balls, pads, & cotton buds	ALL	✓	< 5	$		Bouslimani et al. (2015), Shetage et al. (2014), Sansone-Bazzano et al. (1980), Macdonald (1964), Oh et al. (2014), Jones et al. (1951), Kintz et al. (2000), Sinclair (2019) and Sarkar et al. (2021)
	Medical gauze	ALL	✓	< 5	$		Trivedi et al. (2019), Fu et al. (2022), Jones et al. (1951), Sinclair (2019), Spick et al. (2021), Curran et al. (2005) and Sinclair et al. (2021)
	Tape stripping	Corneocytes	✓	< 5	$$		Ludovici et al. (2018), Shetage et al. (2014), Jacobi et al. (2005), Kim et al. (2010), Cotterill et al. (1972b), Berekméri et al. (2019), Kezic et al. (2009) and Misra et al. (2021)
	Polymeric film (*e.g.,* PDMS, Sebutape®)	ALL	✓	15–60	$$		Shetage et al. (2014), Roh et al. (2006), Piérard-Franchimont et al. (1991), Pappas et al. (2013), Qiu et al. (2011), Piérard-Franchimont et al. (1990), Youn et al. (2005), Verschoore et al. (1993), Fur et al. (2001), Clarys and Barel (1995), Piérard et al. (2000), Bruheim et al. (2003), Riazanskaia et al. (2008), Martin et al. (2014), Jiang et al. (2013), Camera et al. (2010), Lester et al. (2002), Joseph et al. (1998), Pagnoni et al. (1994), Camera et al. (2016), Thomas et al. (2010), Youn et al. (2002), Martin et al. (2016), Roodt et al. (2018), Yin et al. (2022), Ding et al. (2022), Ma et al. (2022) and Li et al. (2022)
	Hydrogels	Polar**	✓	10–20	$$		Dutkiewicz et al. (2014), Dutkiewicz et al. (2015) and Dutkiewicz et al. (2016)
	Ambient ionisation mass spectrometry via probes	ALL		< 5	$		Cho et al. (2021), Hiraoka et al. (2020), Cho et al. (2022), Martínez-Lozano and Mora (2009)
Headspace	SPME fibres	VOCs**		15–30	$$		Bruheim et al. (2003), Zhang et al. (2005) and Duffy et al. (2017)
	Passive flux samplers	VOCs		60–420	$$$		Sekine et al. (2007), Kimura et al. (2016), Furukawa et al. (2017) and Sekine et al. (2018)
	“Wearables” (e.g., PDMS bracelets and anklets*,* nylon socks, silicone wristbands, Teflon sleeves & bags)	VOCs	✓	60+	N/A		Roodt et al. (2018), O’Connell et al. (2014), Robinson et al. (2018), Moraes et al. (2018) and Pulido et al. (2021)

*By “ALL”, the sampling technique aims to collect all sebaceous analytes regardless of origin, chemical, or physical properties, but will have different degrees of bias regarding analyte selectivity that require further study.

**Can be modified to target various metabolite classes or polarities.

Currently, there is no standardised sampling and extraction approach for sebum. There is a growing demand for the harmonisation of analytical methods across research groups to establish quality standards and enable reliable interlaboratory comparison of results. The challenges associated with sebum sampling (and by extension skin surface sampling) have been summarised in Table 3, where the main areas requiring substantial work are sampling reproducibility, standardisation, and understanding population-scale biological variability (discussed previously). Reproducibility is typically seen in research articles through the application of strict in-house sampling conditions by experts, e.g., room temperature, relative humidity, sampling pressure, sampling area, time, and washing, and replicate determinations that allow variation assessments, such as coefficients of variation (%CV).

**Table 3:** A summary of the main challenges associated with skin surface sampling and author-recommended mitigation strategies that can be performed to control them

Challenges with skin surface sampling	Potential mitigation strategies						
	Strict control of sampling conditions	Establishment of endogenous internal markers	Choice and development of sebum sampling technique	Baselining sebum studies	Collection of metadata through questionnaires and medical history	Better understanding of downstream sampling biases	Collection of replicates
Surface roughness that can impact the adhesion of the sampling medium	✓		✓				
Variable contributions from the stratum corneum (corneocyte debris and proteolytic products of filaggrin), sebaceous glands (sebum), and sweat glands (sweat) to the samples	✓	✓	✓	✓	✓	✓	✓
Intra- and inter-variability in sebum composition and sebum production rate	✓	✓		✓	✓		✓
Exogenous contamination from cosmetics, dirt, and pollutants	✓		✓	✓	✓		✓
Sampling and extraction bias introduced during sample preparation		✓	✓			✓	
Reproducibility and repeatability of sample collection	✓	✓	✓				✓

As sebum is lipid-rich, the standardisation of analyte responses post-collection is typically performed through the use of commercially available lipid standards from AVANTI Polar Lipids through the LIPID MAPS initiative to aid quantification and identification (Wenk, 2010); however, a key problem that requires addressing is the standardisation of the sebum collection itself. The inter-variability of sebum production rates means that every individual over a given time and condition will have differing amounts of sebum on their skin. Due to the small volumes collected and the potential simultaneous loss of sampling medium on skin contact, gravimetric determinations of sebum are difficult and most modern studies do not collect this information. This means that sample volume remains uncontrolled during sampling unlike other biofluids, such as blood, urine, or bile. An innovative solution to this would be establishing internal standard markers within sebum that could standardise collection. Key requirements of this molecular marker include specificity to sebum, representation across all populations, stability, and detectability on chosen analytical platforms. To further understand the relative contributions from the sebaceous glands (sebum), sweat glands (sweat), and stratum corneum (corneocyte debris and proteolytic products of filaggrin) for a given sample, internal markers for each can be established. Two papers by Ludovici et al*.* and Michael-Jubeli et al*.* explore the uses of squalene and free fatty acids for sebum markers, and cholesterol and cholesterol sulfate for stratum corneum (Ludovici et al., 2018; Michael-Jubeli et al., 2011). For clinical research, the use of these markers could also minimise false negatives as shown by Ismail et al*.,* who used endogenous sweat markers to verify the suitability of fingerprint sweat collection for tuberculosis treatment adherence purposes (Ismail et al., 2022).

The standardiszation of sebum collection would aid the next steps of baselining sebum, addressing sampling consistency issues, and accounting for exogenous contamination. This is because it would enable the following: (1) normalisation of analyte responses that could better intra- and inter-variability, (2) quantification of metabolites (absolute or semi-quantitative), and (3) assessments of sample and instrument suitability post-analysis, i.e., sensitivity and quality of collection. Sampling and sample quality would become more controlled, which as a result, would give researchers more confidence in their output data and conclusions, particularly for evaluating any outlier results.

The subsequent work required for baselining sebum and addressing exogenous contamination (of all forms, e.g., environmental, cosmetics, dirt, and chemicals) should not be understated as they both require data gathering and/or sharing activities of big data at population scales. This is an area that requires community effort once sebum analyses are more standardised and harmonised to allow interlaboratory collaborative activities, such as the creation of a universal sebum standard and/or a common sebum database. This would help sebum catch up to the level of understanding we have for biofluids such as plasma and serum.

Performing this literature review revealed polymeric films to be the most popular sebum sampling technique, accounting for 25.0% of the total number of publications presented inTable 2.^2^ The merits of this sampling technique include chemical stability, point-of-care potential, and wide sebaceous analyte coverage; however, it is expensive and its incompatibility with some solvents, such as chloroform and dichloromethane, limits the downstream extraction of the sample. Considering sampling speed, cost, and analyte coverage (Table 2), using silica plates and cotton tools could be preferable and can be purchased for less than 1/10 of the cost of commercially available polymeric film. The material integrity and/or pre-washing and activation steps required for absorbent papers, sponges, hydrogels, and gauze make these techniques laborious and less robust in practice (Clarys & Barel, 1995; Cunliffe & Shuster, 1969; Jones et al., 1951; Shetage et al., 2014). Headspace sampling techniques are susceptible to environmental contamination, requiring either cumbersome housing or more rigorous data interpretation.

The non-invasive and superficial nature of sebum sampling makes it suited for both home sampling and wearable technology. Home sampling using pre-packaged kits allows individuals to take a sample at any location, send it to a laboratory for testing, and then receive a result in the next couple of days. Home sampling is not only convenient, easier, and more accessible for patients, but it also provides major cost savings by increasing the testing capacity without major investments in existing services and removing the need of trained personnel to perform the sampling. Prior to this, test manufacturers would need to adequately demonstrate the reliability, stability, and robustness of their chosen sebum biomarkers; their quality should be independent of confounding factors, such as user error, environmental exposure, and variable transport times at different storage conditions. Here, a sebum internal marker could help normalise and check the eligibility of samples in terms of sensitivity and make-up upon receipt. Clear instructions and secure packaging should minimise contamination. Despite this, the improved uptake and patient engagement with home sampling tests should outweigh any performance risks given that clinical performance has been assessed (Tidy et al., 2018). The power of home sampling has been exemplified through both the COVID-19 pandemic (Guglielmi, 2020; Humphreys et al., 2022) and HIV testing (Johnson et al., 2017; Krause et al., 2013; McGuire et al., 2021; WHO, 2020). Sampling via commercially available cotton tools, tapes, and polymeric films are readily adaptable for home sampling. Table 2 shows some ‘wearable’ sampling approaches, such as nylon socks and Teflon sleeves, that have been successful in their target applications, but potentially aspire to future wearable technology. With an increased understanding of sebum and its components, real-time sampling and health monitoring is plausible.

After the sample has been collected, it typically undergoes pre-treatment before analysis, which will introduce further bias. The combined sample collection and its subsequent extraction is a bottleneck in metabolomics due to the large structural diversities of metabolites in biological matrices. Solvent extractions are the gold standard in metabolomics sample pre-treatment. Most of the metabolomics literature uses soluble organic solvents or biphasic liquid–liquid extractions that rely on analyte partitioning between an aqueous and organic phase. The most widely used solvent extraction systems are the biphasic chloroform/methanol/water mixtures, i.e., the “Folch” (Folch et al., 1957) and “Bligh and Dyer” (Bligh & Dyer, 1959) protocols; however, these classical methods are being challenged by new liquid extraction methods, such as the biphasic “Matyash” (Matyash et al., 2008) or “BUME” (Löfgren et al., 2012) protocols, or alternatively monophasic alcoholic solutions using isopropanol, methanol, or ethanol, due to reduced toxicity, costs, and ease of use (Gil et al., 2018; Pellegrino et al., 2014; Sarafian et al., 2014; Satomi et al., 2017; Wong et al., 2019). Further, monophasic methods have greater automation potential, but precaution needs to be taken to avoid precipitation of lipid classes, such as triacylglycerols (Köfeler et al., 2021). Alternative popular pre-treatment approaches that are more targeted include derivatisation, e.g., esterification, silylation, or charge-switch derivatisation, and solid-phase extraction (SPE). Subsequently, the final treated extract is typically subjected to evaporation and reconstitution prior to the introduction to the analytical platform. Modern methods typically employ liquid chromatography or gas chromatography coupled to high-resolution mass spectrometry (HRMS) for untargeted profiling or tandem mass spectrometry (MS/MS) for targeted quantitative analyses. A review of the research articles in Table 2 published after 1999 (n = 60) reveal gas chromatography-mass spectrometry (GC–MS) and reverse-phase liquid chromatography-mass spectrometry (RPLC-MS) to be most popular techniques for sebum analysis, accounting for 43.3% and 23.3% of total publications, respectively. Further discussion of the downstream metabolomics workflow is out of scope of this review but has been reviewed elsewhere (Alonso et al., 2015; Cui et al., 2018; Dudzik et al., 2018; Schrimpe-Rutledge et al., 2016).

An emerging area that avoids the sampling biases imposed by either the sebum sampling technique or the laborious sample pre-treatment mentioned above is ambient ionisation mass spectrometry (AIMS) with/without direct skin analysis. The use of AIMS directly after sample collection by media (Table 2), such as swabs (Bouslimani et al., 2015; Sarkar et al., 2021) or filter paper (Motoyama & Kihara, 2017), skips the need for solvent extraction; whereas, direct skin analysis, using a harmless ionisation technique for skin, allows real-time in-situ analysis of sebum, potentially capturing metabolites in their native, localised environments to low detection limits (Cho et al., 2021; Cooks et al., 2015; Huang et al., 2010, 2011). Both AIMS approaches allow a high sample throughput (≤ 5 s from collection to data generation (Cho et al., 2021; Zhao et al., 2008) but do not allow molecular separation prior to detection. Considering the successes of AIMS for biological skin analyses in forensic science (Justes et al., 2007; Zhao et al., 2008), drug development (Cho et al., 2022; Katona et al., 2011), and cosmetic science (Motoyama & Kihara 2017), as well as continuous developments regarding probes (Fatou et al., 2018; Meisenbichler et al., 2020; Shamraeva et al., 2022) and portable mass spectrometers (Burns et al., 2022; Hendricks et al., 2014; Li et al., 2014; Mulligan et al., 2006), this is an exciting area to watch. For more information, the following reviews are recommended (Feider et al., 2019; Kuo et al., 2020).

## Applications

As sebum coats the skin surface, it continuously interacts with the host and the external environment. This makes it a versatile yet complicated biofluid that simultaneously collects information from multiple angles: (1) a biological snapshot regarding one’s physiological state *inside* the body via endogenous metabolites, (2) impacts of exogenous chemicals *inside* the body via “semi-endogenous” metabolites, and (3) general exposure to exogenous chemicals *outside* the body via exogenous metabolites. Examples across a variety of clinical activities, such as disease diagnostics, treatment monitoring, forensics, and environmental monitoring, are presented in Table 4.

**Table 4:** Selected examples of clinical applications of sebum

Sampling technique	Sample preparation	Analytical platform	Indication	Metabolites	References
Medical gauze	Dynamic headspace trapping on a TenaxTA adsorbent tube prior to analysis	TD–GC–MS TD–GC–ODP	Parkinson’s	Both untargeted and targeted for eicosane, hippuric acid, octadecanal, perillic aldehyde	Trivedi et al. (2019)
	Solvent extraction in methanol	RPLC–MS	COVID-19	Untargeted	Spick et al. (2021)
Q-tips	Paper spray ionisation	DI–IMS–MS	Parkinson’s	Untargeted	Sarkar et al. (2021)
Silica plates	Solvent extraction in methanol	DI–MS	Cystic fibrosis	Untargeted	Esteves et al. (2018)
	Solvent extraction in 1:1 methanol: water	DI–MS	COVID-19	Untargeted	Delafiori et al. (2021)
	Solvent extraction in methanol	DI–MS	Leprosy	Untargeted	Lima et al. (2015)
Polymeric film	PDMS patches in an unpacked thermal desorption tube	TD–GC–MS	Stress	Untargeted	Martin et al. (2016)
	Sebutape® patches prior to solvent extraction in hexane and SPE	GC–MS	Cocaine and codeine use	Targeted cocaine, codeine, and associated metabolites	Joseph et al. (1998)
Tape stripping	D-Squame® patches	RPLC–MS HILIC–MS	Environmental pollution	Untargeted	Misra et al. (2021)
Passive flux samplers	Monotrap® DCC18 trapping media	TD–GC–MS	Smoking and secondhand smoking	Untargeted	Sekine et al. (2018)
Teflon sleeves and bags	Volatiles were collected on HaySepQ adsorbent polymer filters prior to solvent extraction in dichloromethane	GC–MS	Malaria	Untargeted	Moraes et al. (2018) and Pulido et al. (2021)
Hydrogels	Nano DESI ionisation	DI–MS	Psoriasis	Both untargeted and targeted for choline, glutamic acid, phenylalanine, urocanic acid, lactic acid, and citrulline	Dutkiewicz et al. (2016)

*TD * = thermal desorption, *GC* = gas chromatography, *MS* = mass spectrometry, *ODP* = olfactory detection port, *IMS* = ion mobility spectrometry, *RPLC* = reversed-phase liquid chromatography, *HILIC* = hydrophilic interaction liquid chromatography, *DI* = direct infusion, *DESI* = desorption electrospray ionisation

Due to the interface between sebum and blood circulation, sebum analysis has a vast untapped potential for non-invasive biomarker discovery. The identification of a single, unique biomarker specific to a disease is unlikely, and therefore, it is more common to diagnose using a combination of compounds in the form of a fingerprint. For example, by swabbing with medical gauze on the upper back, a ‘compound biomarker panel’ of eicosane, hippuric acid, octadecanal, and perillic aldehyde discriminated Parkinson’s patients from healthy controls using GC–MS (Trivedi et al., 2019). Currently, Parkinson’s disease lacks a clear objective diagnostic test where it relies on a medical history and physical examination, so this breakthrough study highlights the potential to shift to earlier and more accurate detection. Another study concerning malaria that used Teflon sleeves to collect arm and foot VOCs identified biomarker panels of ≤ 22 VOCs using GC–MS to differentiate symptomatic and asymptomatic malaria infection from healthy controls (Moraes et al., 2018). This outperformed the currently available rapid diagnostic tests as it stratified malaria infection to a higher sensitivity, whereby low-level infections would be missed by microscopy. For some diseases, such as COVID-19 (Delafiori et al., 2021; Spick et al., 2021) and leprosy (Lima et al., 2015), biomarkers have been identified in sebum that could replace the swabbing and biopsies currently employed, respectively.

Sebomics has also been applied for drug monitoring and forensic purposes. For instance, Joseph et al*.* collected forehead samples using polymeric film to study the pharmacokinetics of cocaine and codeine in sebum on GC–MS (Joseph et al., 1998). Here, cocaine and codeine administered via subcutaneous injection and oral administration, respectively, were shown to be detected in sebum only 1–2 h after dosing and continued to be detected for 1–2 days. The presence of both drugs in sebum at this rate is a surprising result given that the sebum secretion process takes weeks, highlighting the missing understanding of how analytes transfer across biological matrices, i.e., sebum, sweat, and blood. Another group used agarose hydrogel micro patch-arrayed pads analysed by AIMS to study the spatiotemporal dispersion of topical drugs in vivo, using nicotine and scopolamine for proof of concept (Dutkiewicz et al., 2015). Kintz et al*.* detected cannabinoids in impaired drivers using a cotton pad spiked with water/isopropanol (1:1) on their foreheads with GC–MS, highlighting a potential to move away from urine testing that has an unsuitably long window (several days) for the retrospective detection of illicit drugs to potential rapid roadside testing (Kintz et al., 2000).

An in vitro study using artificial sebum on pig skin confirmed that sebum could theoretically trap organic chemical vapours in vivo after topical exposure (Wakefield et al., 2008). Additionally, Wakefield et al*.* have demonstrated through an in vitro study using artificial sebum that sebum uptakes both benzene and methanol upon vapour exposure (Wakefield et al., 2008). This is suspected to occur through the preferential partitioning of lipophilic environmental chemicals from nature into sebum and therefore, sebum could potentially be used as a human biomonitoring matrix. Misra et al*.* performed multi-omics involving metabolomics by tape stripping women’s cheeks across two Chinese cities of different pollution levels to investigate the effects of environmental pollution on metabolic pathways using RPLC–MS and HILIC–MS. Interestingly, a possible metabolite linked to the air pollutant caprolactam was found in the skin of women exposed to higher pollution levels (Misra et al., 2021), potentially demonstrating sebum’s absorptive properties towards exogenous chemicals. Alternatively, by using headspace-trapping technologies that limit environmental exposure as to create a well-controlled sebum sampling environment, such as passive flux samplers and housed SPME fibres, the metabolic products of skin secretions due to exogenous chemical exposure can be studied. Sekine et al*.* detected numerous smoking-related metabolites in human skin gas compositions of non-smokers exposed to second-hand smoking by GC–MS using passive flux samplers (Sekine et al. 2018).

## Concluding remarks and future perspectives

Metabolomics analyses have a critical role in clinical diagnostics. The reflection of molecular phenotype of humans for disease and health is captured by the measurements of small molecules found in biofluids. The value of a biofluid can be determined by how easy it is to collect and process, what information it holds, how that information can be understood, and what it tells us about the molecular make-up. As shown by its applications across clinical, forensic, and environmental studies, the versatility of sebum for metabolomics lends itself to improving current practices regarding clinical observations and human biomonitoring. Being influenced by both endogenous and exogenous processes, sebum offers a comprehensive, unique fingerprint of an individual’s physiology and environmental exposure.

As a novel biofluid, the major challenge currently limiting its wider application is the lack of standardisation of the sebum collection itself. A lack of quantitative information for the volume collected hinders the meaningful quantitation of the metabolites measured. Consequently, any comprehensive analyte response normalisation to account for errors and any post-analysis sample suitability assessments are lacking. Here, the establishment of endogenous internal markers, such as squalene or select fatty acids for sebum, is a possible solution. This does not slow down the progress of qualitative or semi-quantitative sebum studies, but impedes the progress of quantitative studies, resulting in limited gains addressing the other difficulties associated with skin surface sampling, such as biological variability, sampling consistency, variable contributions from stratum corneum, sebaceous glands, and sweat glands, and finally exogenous contamination. This understanding about sebum and its controlled sampling is mandatory for any future translation of a sebum-based metabolomics workflow to clinical settings, where the sampling procedures are typically distanced from the method developers, i.e., going from in-house metabolomics experts to healthcare professionals, necessitating robust and reliable end-to-end workflows.

Sebum has a huge potential beyond skin research due to currently available metabolomics resources. Understanding how one’s physiological state affects or reflects on the skin metabolome via the sebaceous glands itself or by interactions with sebaceous secretion, will open doors for simpler biomonitoring. Sebum acting as a sink to environmental metabolites has applications awaiting to be explored, such as biosecurity, cross-border migration, localised exposure to harmful substances, and high-throughput population screening. These applications will be possible with rapid advances in volatile headspace and lipidomics method development as well as the ability of the metabolomics community to annotate unknown species better. A key issue with skin surface analysis that remains unsolved is attributing the source of the metabolites found on the skin surface before meaningful biological interpretation.

With the global movement towards home sampling and digital healthcare, particularly emphasised through the COVID-19 pandemic, sebum can accommodate this shift due to its non-invasive, superficial, and readily available nature. Further development in wearable sebum technology, e.g., smart/fitness trackers, is considered likely that could revolutionise not only health and exposure monitoring, but also doctor-patient relationships by providing 24/7 accessible digital data. The implications of this shift to sebum include (but are not limited to) improved remote patient monitoring and personalised care in terms of wearable technology, and increased cost savings, resource efficiency, and patient accessibility by home sampling kits. While laboratory-based analysis is reliable and accurate, with advancement in wearable technologies, biosensors tailored to health monitoring by detection and quantitation of sebum metabolites to extrapolate prognostics are not too distant. We anticipate that developments in this exciting area will deliver novel clinical applications over the next decade.

## Data Availability

No new research data were generated for producing this review. All data derived from cited manuscripts will be made available upon request.

## Footnotes

1 The bibliometric analysis was performed on the Scopus search engine (15th July 2022) using the keywords “serum”, “plasma”, “whole blood”, “faeces”, “urine”, “saliva”, “sebum”, “sputum”, “cerebrospinal fluid”, “ascite”, “cheek swab”, “sperm”, “vaginal discharge”, “bone marrow”, “breast milk”, “mucus”, “bile”, “pus”, “phlegm”, “semen”, “earwax”, “vomit”, “amniotic fluid”, “gastric acid”, “exudate”, “aqueous humour”, “cerumen”, “chyle”, “lymph”, “pericardial fluid”, “peritoneal fluid”, “pleural fluid”, “serous fluid”, “synovial fluid”, and “menstrual fluid” followed by “AND “metabolomics”” for each. The search was limited to papers published in 2021. A percentage was calculated by dividing the number of publications for each keyword search by the total number of publications of all the keyword searches combined, then multiplying by 100. Here, the term “blood” combines the percentages calculated for the keyword searches of “serum”, “plasma”, and “whole blood”.

2 The references in Table 2 are, to the best of our ability, exhaustive of all sebum or skin metabolomics analyses (not including sweat or stratum corneum analysis) found across the Web of Science, Scopus, and Google Scholar search engines (12th January 2023). A percentage was calculated by dividing the number of publications for polymeric film by the total number of publications of all skin analyses reported in Table 2, then multiplying by 100.
